# Immunological events in chronic spontaneous urticaria

**DOI:** 10.1186/s13601-015-0074-7

**Published:** 2015-08-25

**Authors:** Marta Ferrer

**Affiliations:** Department of Allergy and Clinical Immunology, Clinica Universidad de Navarra, Pio XII, 36, 31008 Pamplona, Spain

## Abstract

Chronic spontaneous urticaria (CSU) is a highly debilitating skin disease associated with systemic features. We have made significant progress in several aspects relating to this condition. However, the exact physiopathology remains unknown. There is mounting evidence for an autoimmune basis, demonstrated by the CSU serum ability to activate healthy donors skin mast cells and blood basophils. However, it is only seen among 35–40% of patients. Mast cells and basophils play an important role in this skin condition. Both cells in CSU patients have unique features that differentiate them from basophils and mast cells from healthy donors. In the case of basophils, basopenia is typically found in CSU patients. Basophils from CSU patients also tend to be hyporesponsive to stimuli that act through the IgE receptor, responsive to other stimuli as MCP-1 or C5a, and hyperesponsive when incubated with sera. Eosinophils are also present in CSU skin biopsies, yet their exact role has not yet been defined. Likewise, endothelial cells also play a function, as indirectly demonstrated by an increase of vasoactive peptides in skin and plasma of CSU patients’ samples. All these facts orchestrate a systemic inflammation response producing a significant increase of several inflammatory markers. Unfortunately, we lack a unitary model that could explain the exact role of each of these players. In this review, we will describe the history and discover the pathway to the present knowledge on the immunological facts of this disease.

## Introduction

Chronic spontaneous urticaria (CSU) consists of the daily appearance of pruritic wheals angioedema or both [1] for more than 6 weeks. Since the first description recorded in history in the 10th century BC in China [2], we have greatly improved our clinical approach, severity scoring, quality of life assessment, as well as tools to control the disease. However, we are still far from understanding the physiopathology of the disorder. In this review we will briefly narrate the history of the developments that have led to a potential explanation of the immunology underlying CSU.

## Autoimmunity

It was in 1946 when for the first time Malmros [3] conducted for the first time his Autoserum test among 956 patients with many different types of disease, likened the wheal and flare to a histamine reaction, reporting that some patients with CSU (6 out of 53 positive) gave a positive autoserum test. This finding remained overlooked for another 47 years until Leznoff [4] noted a 15% prevalence of autoimmune thyroid antibodies in patients suffering from CSU but with a normal thyroid function. Other authors have confirmed these findings [5, 6]. This is the reason why thyroid antibody determination can be a useful tool as an indirect marker for autoimmunity.

In 1986, Grattan [7] reported that the serum from 12 patients suffering from active CSU were able to induce a positive autologous skin reaction. Subsequently, Gruber and Kaplan [8] demonstrated a non-functional IgG anti-IgE by an immunoassay. Only one patient with cold urticaria showed a functional IgM anti-IgE. Later on, Grattan was the first to describe histamine releasing autoantibodies with functional properties of anti-IgE in CSU [9], this paper set the scene for the subsequent discoveries. The next big step was made when M Greaves and co-workers [10] demonstrated for the first time the functional autoantibodies in CSU. CSU sera were indeed able to activate basophils from normal donors. This ability was increased if the IgE bound to the basophils was removed with lactic acid and decreased when basophils were incubated with human IgE that occupies the IgE receptor. These authors also found that in 20% of patients, IgE was needed to activate the basophils. In connection with this, they deduced that they might have anti IgE antibodies. After [11] they demonstrated mast cell degranulation upon incubation with sera from a group of 163 CSU patients. Moreover, they also obtained histamine release in IgE- and non-IgE-sensitized basophils. They detected anti-FcεRI α antibodies in 25% of CSU sera and to a lesser extent anti IgE antibodies. Notably, those sera that induced basophil histamine release did so with mast cells. Fiebiger and coworkers discovered that purified IgG from a subset (37%) of CSU patients was able to bind to the IgE receptor. They also compared IgG from patients with atopic dermatitis and healthy donors and none of them demonstrated such an ability [12]. Furthermore [13] similar antibodies in other skin autoimmune diseases were also noted but these corresponded to IgG2 or IgG4 and were not functional, whereas in the case of CSU the antibodies corresponded to IgG1 and IgG3. Interestingly, only IgG1 and IgG3 subtypes are able to activate complement.

This hypothesis was confirmed by Kaplan [14] who transfected rat leukemia basophils with the α subunit of the IgE receptor and confirmed that such activation was through the interaction with the IgE receptor. An indirect observation of mast cell degranulation upon autologous serum injection [15] also confirmed that the antibodies against the α subunit of the FcεRI receptor have the ability to induce mast cell degranulation.

Finaly, Kinet sequenced and cloned the α subunit of the IgE receptor and demonstrated [16] through immunoblotting that IgG from CSU sera recognized this fraction. This ability was not found in the control sera in any instance. They also were able to demonstrate that IgG and CSU sera were also able to activate mast cells with exactly the same 50% frequency as found in basophils.

Nevertheless, there is no correlation between serum reactive IgG antibodies across functional studies, Western-blot analyses and autologous skin tests [17]. The reason is still unknown. The cause is multifactorial. The high variability in basophil response to patient sera, the IgG subclass distribution and the number of α subunits occupied as well as conformational or glycosylation differences between the cloned and human α subunit are the associated factors.

In 2001 Horn [18] using intravenous immuno globulin samples, found Fc-ε α antibodies present in healthy donors that cross-react with tetanus toxoid. However, when Ferrer and co-workers attempted (data not published) to inhibit the binding to the α subunit by preincubating sera with tetanus toxoid, were not able to demonstrate such inhibition. Moreover, these data were not further confirmed. Using a tonsilar IgM library this group also found [19] antibodies against the α subunit of the IgE receptor that were able to induce histamine release in healthy donors. However, in order to induce such degranulation, removal of the already bound IgE was required. The authors hypothesized that these might represent conditional antibodies that at some point could become pathogenic because those antibodies could bind cutaneous mast cells if IgE dissociated from FcεRI due to local conditions, thereby eliciting a local wheal. The existence of such antibodies has not yet been independently confirmed.

As was previously mentioned, the IgG1 or IgG3 subtypes were the subclasses identified in CSU. Utilizing purified IgG and decomplemented sera, in a study performed by Ferrer et al. were able to demonstrate that mast cell and basophil histamine release were dose-dependent on C5a and were inhibited when blocked with a C5a antibody receptor [20]. The authors conclude that the release of histamine by the antibodies against the α subunit of the IgE receptor was increased by C5a activation [21]. The mechanism on how C5 amplifies mast cell activation, as Kaplan points out [22], takes place when two IgG molecules bind to two α receptors, thus activating C5a that consequently activates mast cells. The mechanism for generating C5a by immune complex formation is more likely to be by two anti-FcεRI IgG molecules binding adjacent FceRI on mast cells or basophils. Theoretically, it could also occur as a consequence of complexes IgG anti-IgE bound to its receptor. This would also explain why CSU patients with anti IgE receptor antibodies do not show respiratory nor systemic symptoms.

Furthermore, this ability of CSU serum to activate normal basophils was demonstrated in different ways such as by inducing CD203c [23] or CD63 [24] basophil expression upon incubation with urticaria sera. Both markers are correlated with histamine release. However, not only preformed mediators such as histamine were induced, but also non-preformed mediators as leukotrienes [24] thus indicating that such an ability activates both proximal and distal signalling pathways. Moreover, Ferrer et al. demonstrated that basophil activation also induces the production of cytokines such as IL4 [25] which explains the inflammatory milieu found in CSU biopsies.

Determinations of the HLA class 2 alleles [26] also showed an increase in the HLA DRBI*04 (corrected p = 3.6 × 10^−6^) in patients with positive serum basophil histamine releasing activity and autoreactivity or both, and a favorable association (which was statistically significant) between HLA B*50 [27] and patients with CSU, with an OR (95% CI) of 2.96 (1.17–7.48) consistent with an autoimmune basis.

A very interesting review of the evidence supporting autoimmunity can be found elsewhere [28].

From an epidemiological point of view, a particularly interesting study was carried out by Confino [29] who after following-up a very large CSU population for 10 years, demonstrated a high association between the presence of autoimmune diseases and CSU.

On the basis of all these studies, it is clear that between 35 and 40% antibodies against the α subunit of the IgE receptor and between 5 and 10% react against IgE.

Finally, an indirect evidence in favour of an autoimmune mechanism in a subset of CSU patients is the beneficial response to immunosuppressive agents and immunomodulatory treatment such as cyclosporine [30, 31], plasmapheresis [32] as well as intravenous gamma globulin [33].

Many questions remain open regarding the role of autoimmunity which is the most prominent underlying mechanism of the remaining 40–50% of patients in whom no autoimmunity is found but whose clinical features are identical.

## Mast cells

Mast cells along with basophils play a key role in the pathophysiology of the disease. The first studies reported a significant increase in mast cells in CSU skin lesions [34, 35]. However, use of specific anti-tryptase antibodies did not reproduce this finding [36]. Recently, Kay [37] reviewed this issue finding as was first reported an increase of mast cells in both affected and unaffected CSU skin.

As for mast cell functional features, one of the first studies found that [38] CSU mast cell responses to skin stimuli such as codeine were one hundred times more sensitive than normal control skin mast cells. An interesting finding reported by Jacques and co-workers [39] pointed out that the levels of histamine measured in lesional skin suction blisters correlated with disease activity. Likewise, mast cells cultured from peripheral CD34+ cells from patients with CSU showed greater spontaneous histamine release, increased Syk, and decreased SHIP-2 levels as compared with normal donors [40]. Ferrer and Schwartz [41] studied tryptase levels and found higher tryptase levels in CSU patients as compared with healthy controls and atopic donors, and also in CSU symptomatic patients at the time of the sample collection as compared with asymptomatic patients. However, when they analysed which type of tryptase was elevated, contrary to expectations, this increase was not due to mast cell degranulation and did not find a rise in mature tryptase levels.

One of the most interesting studies dealing with the connection between mast cells and autoimmunity was done by Bossi [42] who cultured two mast cell lines, one expressing IgE receptor (LAD2) and the other not expressing this receptor (HMC-1). Degranulation in both mast cell lines following incubation with CSU sera was found. Moreover, the supernatants of such cultures were able to induce vascular leakage from endothelial cells. This fact highlights the importance of endothelial cells and explains the increased expression of endothelial cell adhesion molecules [43–45]. This issue clearly needs further investigation. As Kay [43] recently reported, there is an increase in vasoactive peptides in lesional CSU skin samples. Kasperska-zajak [46] also found similar data in plasma from CSU patients.

## Basophils

Although the exact role of basophils in this disease has not been demonstrated, it has been shown that peripheral blood basophils from CSU patients have unique features that reverse upon remission and in response to therapy.

A basophil abnormality is of particular interest because some patients with chronic urticaria have basopenia. This was reported as early as 1962 by Rorsman [47] and confirmed later by Grattan [48, 49] who found a correlation with urticaria severity, and was again confirmed later by Eckman [50, 51]. Interestingly, Rorsman attributed the low number of basophils to the presence of an antigen antibody reaction that leads to basophil degranulation, thus suggesting an autoimmune type of disease. The reason for such basopenia is partially explained because the presence of basophils in lesional and non-lesional skin of CSU patients and not in normal skin [52–54] has been demonstrated.

CSU basophils are different not only in number but also in function. In fact, in 1974 Greaves showed that basophils of patients with CSU were hyporesponsive to anti-IgE, and at that time attributed this feature to in vivo desensitization [55]. This quality was later confirmed by Kern and Lichtenstein [56] they reported that this feature was not observed when stimulation was applied using stimuli other than anti-IgE nor was it due to differences in histamine content. In a group of 26 patients, Sabroe correlated the basophil number, presence of autoantibodies and histamine release upon stimuli with anti-IgE and found a more marked basopenia and hypo responsiveness to anti-IgE in the autoimmune group. She postulated that this was due to basophil desensitization of the FcεRI pathway [57].

Ferrer compared [25] the response of basophils of healthy donors, atopic donors and CSU patients to a variety of stimuli including anti-IgE, bradykinin, MCP-1, C5a, and serum, and found that the basophils of CSU patients exhibited a diminished response to anti-IgE, and to a lesser degree to C5a. No difference was observed when basophils from CSU patients were incubated with bradykinin or MCP-1. These results are not due to a variation in histamine content since no significant differences was found between the healthy control with the urticaria basophils. Although basophils of chronic urticaria patients seem to be less responsive to stimuli such as anti-IgE or C5a which act through different receptors. The abnormality does not seem to be due to a general impairment of signaling since CSU basophils do respond normally to other stimuli that act independently from the IgE receptor such as PAF [58] in addition to bradykinin [59], and MCP-1 [60].

Surprisingly, Ferrer also observed [61] prominent histamine release when basophils of CSU patients were stimulated with other sera regardless of source. Thus, marked histamine release was obtained with sera derived from patients with chronic autoimmune and non autoimmune urticaria, or even from the normal control sera. The factor in serum that stimulates these cells has not been identified nor is the abnormal responsiveness of the cells understood. But it is clearly specific for CSU since it has never found when incubating serum with healthy normal or atopic basophil donors.

Basophils derived from patients with non autoimmune CSU were just as abnormal as basophils from patients with chronic autoimmune urticaria. Both groups of basophil were equally responsive to bradykinin, C5a, MCP-1, or serum. Both groups were also hyporesponsive to anti IgE, thus in vivo desensitization due to the presence of autoantibody does not seem to be the explanation. The abnormality of signal transduction remains to be defined. Likewise, Lourenço [62] also found an increased surface FcεRI expression in the basophils of patients suffering from CSU and increased response to IL3. However, adding IL3 does not change those non releasers sera into releasers [63].

Interestingly, a recent paper by Saini [64] offered additional insight into this issue. The authors demonstrated the ability of active CSU serum to transfer FcεRI-mediated basophil histamine release suppression to healthy basophils. They did so by culturing healthy basophils with sera from patients with CSU and found a marked suppression of FcεRI-mediated histamine release compared with media or media with autologous serum. Again, this feature was reversed upon CSU remission. Interestingly, it was not overturned by heating serum (that would remove IgE), IgG or omalizumab.

## Eosinophils

In spite of being the most abundant cells in CSU skin biopsies, little attention has been devoted to eosinophils. One very interesting paper [65] using a peptide library found that CSU patients had IgG against CD23 present on the FcεRII. This is a very attractive discovery since basophils could become activated through eosinophil major basic protein. Unfortunately, no further research has been dedicated to this hypothesis.

## Th1 or Th2 phenotype

CSU is characterized by a perivascular infiltrate surrounding small venules with a predominance of CD4+ T lymphocyte cells [35] along with neutrophils, mast cell basophils, and eosinophils. There is also an increase in vascular markers both in lesional and in non lesional skin [37]. Because of level of inflammatory markers [66, 67], it is a systemic inflammatory disease not only confined to the skin.

The cellular infiltrate resembles the on which can be observed in the late phase allergic reactions. For that reason, Ferrer and co-workers questioned [25] whether the immunologic profile reflects the predominance of a Th1 or Th2 phenotype. They initially approached this objective by determining the cytokine profile in the sera of patients with chronic urticaria. They measured INFγ as a representative of a Th1 profile, and then measured IL4 and IL5 as representatives of a Th2 subtype. They found that IL4 was higher in the sera of patients with chronic urticaria (as well as in atopic subjects) compared to controls while IL5 and IFNγ levels were normal. Significant differences were found in the ability of CD4+ lymphocytes to produce IL4 and INFγ upon PMA-Ionomycin stimulus when healthy controls were compared to chronic urticaria patients. There was no difference in IL4 or INFγ production by CD8+ lymphocytes of patients vs. the control group. Likewise, no significant differences were found when comparing the ratio of INFγ/IL4 production by CD4+ or CD8+ lymphocytes of controls and urticaria patients. The cytokine profile found in our study does not reflect either a Th1 or Th2 predominance.

These data again strengthen an immune basis for chronic urticaria, once demonstrated that the CD4+ lymphocytes of patients with this disease are activated and they release greater amounts of cytokine with a non-specific stimulus. On the other hand, this finding is similar to a study in which the cellular infiltrate associated with chronic urticaria had a Th0 profile [52]. These authors analyzed skin biopsies from chronic urticaria patients by in situ hybridization. IL4, IL5 and INF-gamma revealed higher cytokine m-RNA expression in chronic urticaria patients than in healthy controls, without a predominance of either a Th1 or Th2 representative cytokine.

In a study composed by eight patients analyzed with immunohistochemistry, Kay reported a predominance of Th2 initiating cytokines (IL33, IL25 and TSLP) in skin lesions among patients with CSU [68]. However, it should be noted that IL33 in some instances could also promote a Th1 response [69].

## Coagulation pathway

Recently Asero has reported the activation of the extrinsic coagulation pathway in patients with CSU suggesting that the coagulation cascade might be involved. It was first shown that several markers such as the prothrombin fragment F1+2 [70] and activated factor VII [71] were increased. This could be explained because in cases of severe CSU, the activation of the coagulation cascade could lead to fibrinolysis leading to an increase in D-dimer levels [72]. Interestingly, D-dimer levels correlate with the severity and could predict the lack of response to antihistamines [73]. However, increased D-dimer levelwith disease activity is not specific to CSU since it is also seen in bullous pemphigoid [74] and hereditary angioedema [75]. Thus, it is not specific for mast cell mediated disease.

Moreover, other coagulation cascade proteins are able to directly activate mast cells, as it is in the case of thrombin that cleaves protease-activated receptors [76] 1 (PAR-1) and Tissue Factor plus factor VIIa through PAR-2 [77].

**Fig. 1:**
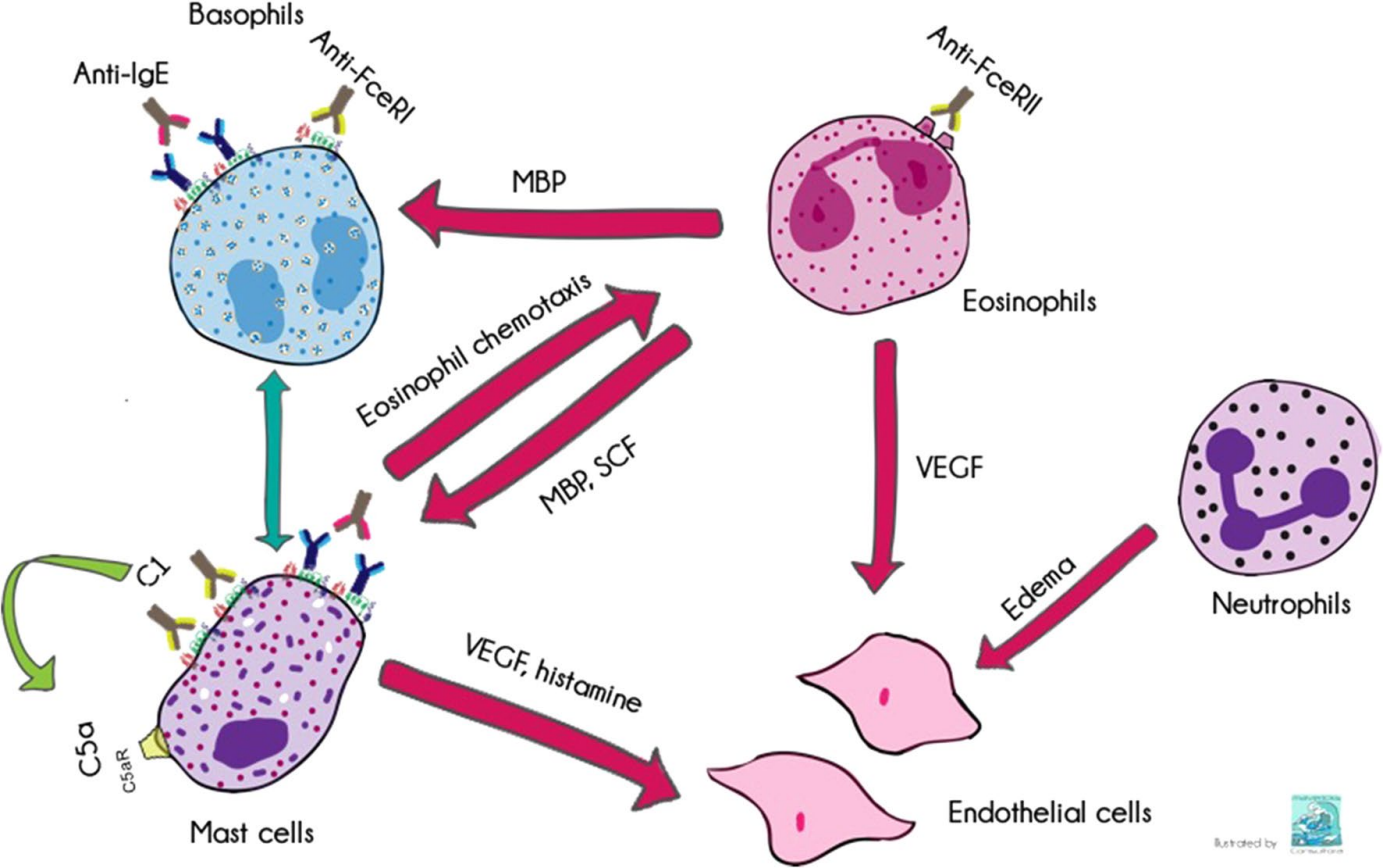
We represent in a very simplistic cartoon the possible ways of activation among cells implicated in this disease. Cross-talk between mast cells and basophils remain to be defined.

The real significance of these facts in the pathogenesis of CSU is not well understood. However, they could amplify the inflammation inducing vascular permeability. Furthermore, some cascade proteins are able to induce mast cell degranulation (we include a summary of these pathways and cell cross-talk in Fig. 1).

## Conclusion

Chronic spontaneous urticaria is an inflammatory disease. There is no single cell type that is uniquely responsible for CSU. Rather, there are several cells involved and each has its own unique role. So far, we have been able to describe different aspects that point in different directions but we lack a unified explanation or a chain of facts that can explain the final outcome. In this regard, several perplexing facts occur. The way corticosteroids are able to control an urticaria flare-up without being able to inhibit mast cell degranulation, or what is even more surprising, the ability of omalizumab to rapidly control the disease by means of a mechanism of action which remains elusive.
